# One-stage repair of proximal hypospadias by in situ tubularization of the transverse preputial island flap

**DOI:** 10.1007/s00345-023-04296-0

**Published:** 2023-02-06

**Authors:** Yiqing Lyu, Fang Chen, Hua Xie, Yichen Huang, Min Wu, Xiaoxi Li, Yan Liang, Zhiwei Peng

**Affiliations:** Department of Urology, Shanghai Children’s Hospital, School of medicine, Shanghai Jiao Tong University, Shanghai, 200040 China

**Keywords:** Hypospadias, One-stage surgery, Pediatric urology, Complication

## Abstract

**Purpose:**

This study aimed to compare the efficacy of modified transverse preputial island flap (TPIF) repair with the traditional TPIF procedure and Byar’s two-stage procedure in proximal hypospadias repair, especially in the postoperative urethral stricture incidence rates.

**Materials and methods:**

Patients admitted for proximal hypospadias treated with modified TPIF repair, the traditional TPIF procedure, or Byar’s two-stage procedure at our institution from 2017 to 2021 were identified, and the incidence of postoperative complications among them was compared.

**Results:**

In total, 142 patients were included (modified TPIF group, 43; traditional TPIF group, 37; and Byar’s two-stage group, 62). The length of the neourethra was 4.21 ± 0.63 cm in the modified TPIF group, 4.18 ± 0.71 cm in the traditional TPIF group, and 4.20 ± 0.68 cm in the Byar’s two-stage group. The rate of urethral stricture in the modified TPIF group (two cases, 4.65%) was significantly lower than that in the traditional TPIF group (four cases, 10.81%) (*P* = 0.008). Seven (16.28%) cases of urethrocutaneous fistula occurred in the modified TPIF group, six (16.22%) in the traditional TPIF group, and eight (12.90%) in the two-stage group. Additionally, one case (2.33%) of urethral diverticulum occurred in the modified TPIF group, one (2.70%) in the traditional TPIF group, and three (4.84%) in Byar’s two-stage group.

**Conclusions:**

Modified TPIF repair can ensure a wedge anastomosis between the proximal urethral meatus and the neourethra, provide support and blood supply for the neourethra. Furthermore, it extended the urethral plate width at the anastomosis and urethral meatus, effectively reducing the incidence of urethral strictures.

## Introduction

Proximal hypospadias poses a significant challenge to urologists. It has spawned nearly a 100 surgical methods over time. However, the prognosis of patients with proximal hypospadias remains unsatisfactory compared to distal hypospadias [1, 2]. Although many surgeons use a two-stage procedure to improve the success rate, several studies have shown that the outcome of a two-stage procedure is not necessarily better than that of a one-stage procedure [3, 4]. Specifically, a one-stage procedure can effectively shorten the treatment period and reduce the economic and psychological burden on patients and families. Therefore, it is still the direction pursued by many surgeons. Among the numerous and sophisticated surgical methods, transverse preputial island flap (TPIF) repair (Duckett’s procedure) is considered one of the most classic and effective surgical procedures. However, the high prevalence of complications after TPIF, particularly urethral stricture, prevents its widespread use [2, 5].

**Fig. 1:**
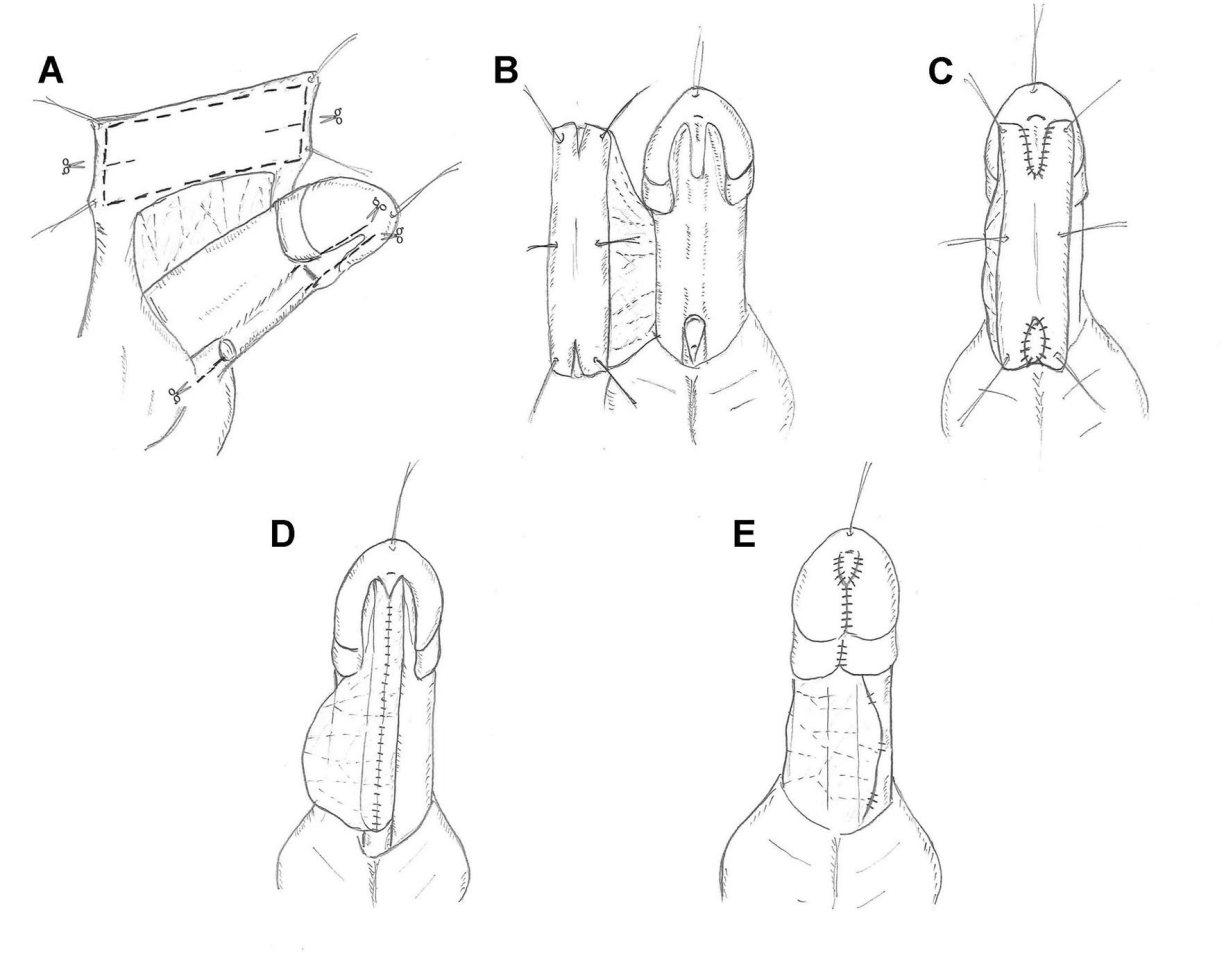
Schematic diagram of the modified TPIF. **A** A transverse pedicle flap is mobilized from the inner prepuce. Several incisions are made along the dot lines. **B** Eventually, V shaped incisions are made at the two ends of the flap. The original urethral meatus is trimmed and spatulated. The bilateral glanular wings were dissected away from the corpora cavernosa. **C** The two ends of the flap are aligned and anastomosed with the urethral plate distally and the native urethra proximally. **D** The flap is tubularized with the suture line facing ventrally. **E** The flap pedicle is draped over the ventral aspect of the neourethra and tacked in place covering the urethral suture line. The bilateral glanular winds are re-approximated covering the neourethra

To this end, since 2017, we have modified the TPIF repair: first, ventrally reconstructing the urethral plate by flap on the penis, followed by tubularizing in situ to construct a neourethra, to reduce postoperative complications, especially urethral stricture (Fig. 1) [6]. After three years of application, we compared this method with the traditional TPIF and Byar’s two-stage procedure and report the results below.

## Materials and methods

A retrospective and IRB-sanctioned study were performed on all patients with proximal hypospadias at the current institution between January 2017 and December 2019. All patients undergoing modified TPIF repair, traditional TPIF procedure, and Byar's two-stage procedure were classified into corresponding groups.

Inclusion criteria: ① Untreated proximal hypospadias; ② Repair with only the modified TPIF, traditional TPIF, or Byar’s two-stage procedures.

Exclusion criteria: ① previous history of hypospadias repair; ② combined repair with other surgical procedures; ③ patients with androgen insensitivity syndrome or unexplained sexual dysplasia.

A total of 43 patients were enrolled in the modified TPIF group, 37 in the traditional TPIF group, and 62 in Byar’s two-stage group. All operations were performed by attending physicians with more than 10 years of experience in hypospadias repair. A comparison of the demographics in each group is shown in Table 1.

**Table 1:** Cohort demographics in each group

	Midified TPIF procedure	Traditional TPIF procedure	Byar’s procedure
Number of cases	43	37	62
Age at surgery (months)	14.25 ± 4.31	14.88 ± 5.05	13.78 ± 4.16
Weight (Kg)	10.25 ± 1.05	10.54 ± 0.98	10.22 ± 1.06
Length of urethral defect (cm)	4.21 ± 0.63	4.18 ± 0.71	4.20 ± 0.68

### Technique

After the skin was degloved, an artificial erection was performed to determine the point of maximal curvature where the urethral plate was transected. Then the urethral plate was divided into distal and proximal part, and both parts were dissected to each end from the transection point along the surface of corpora cavernosa. Any fibrous bands tethering the penis were removed. If a slight curvature remained, a dorsal plication was performed to ensure penile straightening. Next, the glans wings were mobilized according to our glanuloplasty method [7] along the bilateral distal urethral plate to the top of the glans, completely exposing the distal urethral plate.

**Fig. 2:**
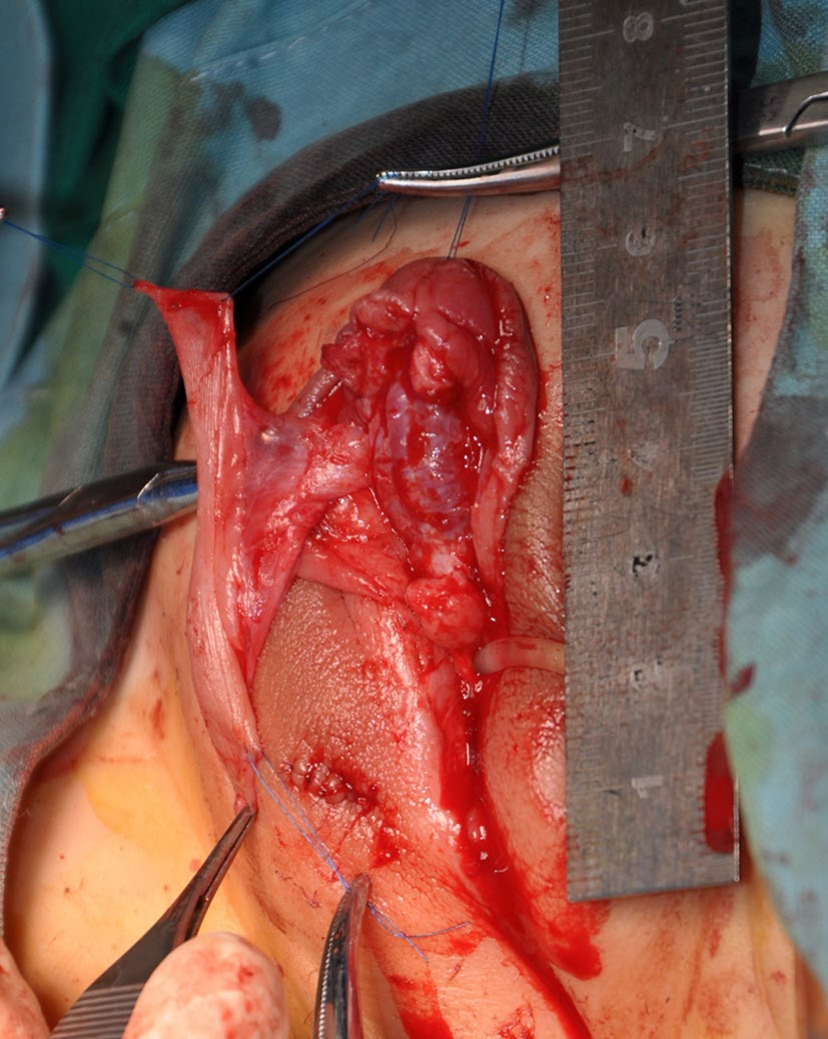
A transverse pedicle flap is mobilized from the inner prepuce

**Fig. 3:**
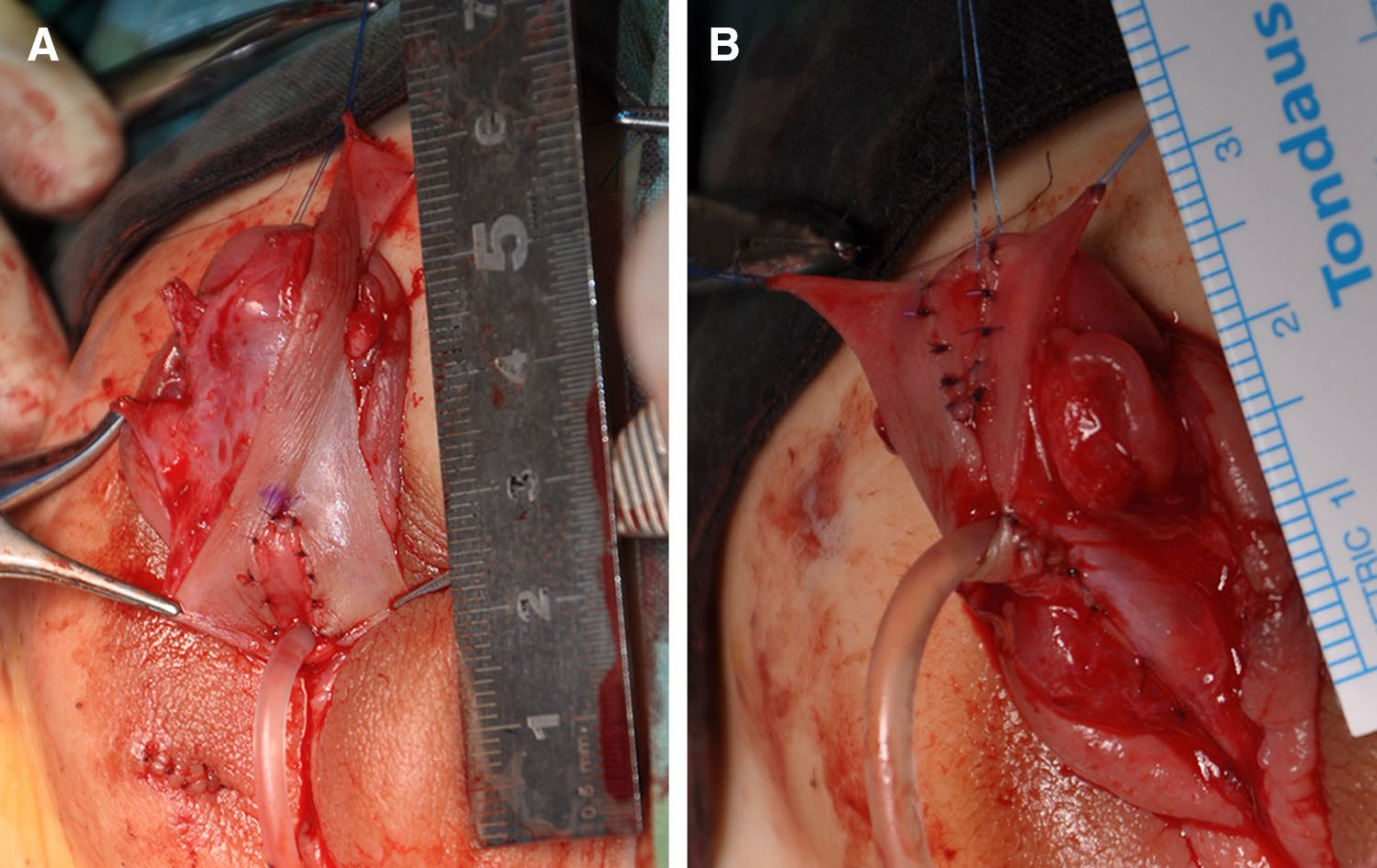
**A** The proximal native meatus is anastomosed with the flap. **B** The distal ends of the flap is anastomosed with the urethral plate at the glan

**Fig. 4:**
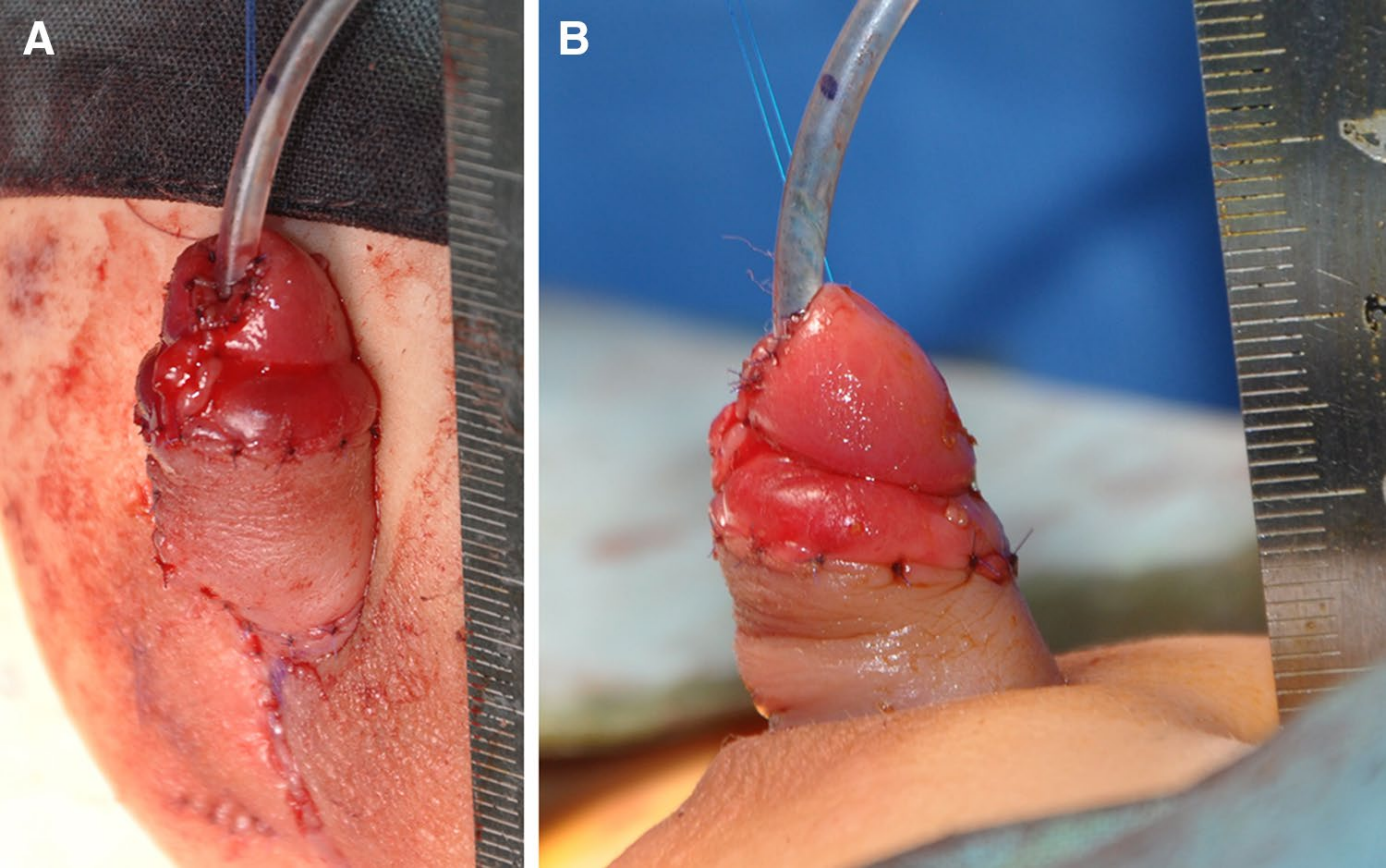
**A** Appearance of the glan and the neo-meatus. **B** Appearance of the penile

Next, a pedicle flap harvested from the dorsal prepuce with a width of 1.5–1.8 cm was transposed directly on the ventral surface of the penis after calculating the length of the urethral defect (Fig. 2). The proximal and distal ends of the flap were trimmed into a V-shape and anastomosed with the native meatus to ensure spoon-shaped anastomosis in both junctions (Fig. 3A, B). An 8 Fr feeding tube was used as a template. The flap was then trimmed and tubularized proximally to distally to construct the neourethra. Subsequently, Darto’s fascia was covered on the neourethra, followed by glanuloplasty, which combined the bilateral distal corpus cavernosum and the glanular wings in the midline. Finally, the preputial skin was tailored to provide adequate coverage of the penis (Fig. 4A, B).

### Postoperative management and follow-up

Prophylactic antibiotics were administered routinely. The catheter was retained until it was removed three weeks postoperatively. Postoperative follow-up was conducted in all patients, from once every 3 months to once every 6–12 months after one year. Complications such as urethrocutaneous fistula, urethral stricture, and urethral diverticulum were the primary prognostic outcomes.

### Statistical analysis

The chi-square test was used to compare the probability of complications between the modified TPIF group and the other two groups. Statistical significance was set at *p* < 0.05, and statistical analyses were performed using SPSS 22.0 (SPSS Inc., Chicago, IL, USA).

### Ethics statement

This study strictly complied with the Declaration of Helsinki and was approved by the Ethics Committee of the Shanghai Children’s Hospital (No. 2019R028-F01). All participants and their families provided informed consent.

## Result

The length of the neourethra was 4.21 ± 0.63 cm in the modified TPIF group, 4.18 ± 0.71 cm in the traditional TPIF group, and 4.20 ± 0.68 cm in the Byar’s two-stage group, respectively. No significant differences in the length of the reconstructed urethra were found between the groups (*P* = 0.087).

The average follow-up time in each group was 27.72 ± 10.19 months (12–63 months). The complications in each group are summarized in Table 2.

**Table 2:** Postoperative complications in each group

Complications	Group	Number of cases, *n* (%)	*P* (compared with the midified TPIF repair)
Urethral stricture			
	Midified TPIF	2 (4.65)	
	Traditional TPIF	4 (10.81)	0.003
	Byar’s	2 (3.23)	0.040
Urethrocutaneous fistula			
	Midified TPIF	7 (16.28)	
	Traditional TPIF	6 (16.22)	0.886
	Byar’s	8 (12.90)	0.038
Urethral diverticulum			
	Midified TPIF	1 (2.33)	
	Traditional TPIF	1 (2.70)	0.753
	Byar’s	3 (4.84)	0.029

Of the 43 patients in the modified TPIF group, 2 (4.65%) developed glanular urethral strictures postoperatively, with an original glanular width of less than 8 mm. One case was relieved by dilation, and the other was alleviated by incising the glanular urethra from the urethral meatus to the level of the coronary sulcus. There were four (10.81%, *P* = 0.003) cases of urethral stricture in the traditional TPIF group, one at the level of the coronary sulcus and three at the proximal anastomosis. All four patients underwent urethrostomy and resection of the stricture segments, followed by second-stage reconstruction of the urethra. In the two-stage group, two patients (3.23%, *P* = 0.040) developed urethral strictures in the proximal anastomosis. These were also relieved by urethrostomy, resection of stricture segments, and second-stage reconstruction of the urethra.

Seven cases (16.28%) of urethrocutaneous fistula were postoperatively identified in the modified TPIF group. Of the seven cases, two occurred at the coronary sulcus, four at the penile body, and one at the penile root. On the other hand, there were six (16.22%, *P* = 0.886) cases of urethrocutaneous fistula in the traditional TPIF group, including one case combined with coronary stenosis. Of these six cases, two occurred at the coronary sulcus, three at the corpus penis, and one at the radix penis. As for Byar’s two-stage group, eight (12.90%, *P* = 0.038) cases developed urethrocutaneous fistulas, three at the coronary sulcus, three at the corpus penis, and one at the radix penis.

Urethral diverticulum was rare in this cohort. One (2.33%) case occurred in the modified TPIF group, one (2.70%, *P* = 0.753) in the traditional TPIF group, and three (4.84%, *P* = 0.029) in Byar’s two-stage group. No diverticula were accompanied by stricture or urethrocutaneous fistula. All cases recovered after re-urethroplasty by trimming the diverticulum.

## Discussion

Compared to distal hypospadias, proximal hypospadias represents a spectrum of external genitalia malformations. These include high degrees of penile curvature, long urethral defects, dysplastic glans, and penoscrotal inversion, which lead to more repair and plastic reconstruction. Both short-term and long-term outcomes are usually accompanied by a high prevalence of postoperative complications.

It was not until the 1980s, when Professor Duckett proposed using TPIF, a historical innovation, to reconstruct the urethra [8]. This has several advantages, such as completely straightening the penis, convenient access to materials, and a one-stage repair. Subsequently, the one-stage TPIF gradually replaced the former two-stage procedure and became the direction pursued by urologists. Nevertheless, later practice verification conducted by urologists found that this technique was accompanied by the shortcomings of a long learning curve and various complications. Although experienced surgeons improve their proficiency and make various modifications through long-term practice, the complication rate remains as high as ~ 37.9% [5, 9, 10]. This could be explained by complex surgical procedures, abnormal distribution of foreskin, increased infection risk, and the surgeon’s proficiency and experience requirements. Therefore, the concept of two-stage repair has been proposed and has attracted considerable attention. Currently, less than 40% of surgeons choose a one-stage procedure for proximal hypospadias.

The two-stage procedure is inferior to the one-stage procedure in terms of the treatment period, cost, anesthesia risk, and psychological burden. Consequently, if the high prevalence of complications in the TPIF procedure can be improved, it remains a clinically effective technique that can significantly benefit patients. Among all the complications of the TPIF procedure, the urethral stricture is a severe complication with disastrous consequences and a high incidence rate of approximately 12.5% [5]. Once it occurs, it is difficult to relieve by dilation and usually leads to a failed repair in severe cases, even the “re-do” surgery or crippled penis. This type of urinary stricture primarily exists at the site of the glanular urethra and anastomosis with the proximal urethral meatus [9, 11]. Anatomically and technically, although Duckett requires that the proximal anastomosis be made into a spoon shape, it is difficult to achieve when trimming the flap into a tube, causing the diameter of the anastomotic urethra to be narrower than that of the adjacent urethra. In addition, the glanular neourethra presents a tunnel through dense granular cavernous tissue with an external pressure significantly higher than that of the penile urethra and scrotal segments. In addition, the distal urethra is also the peripheral part of the blood supply to the flap, resulting in a high probability of urinary stricture caused by an insufficient blood supply. To solve this problem, the tube diameter and blood supply at both anastomoses of the neourethra appear to be the focus.

The urethral plate has long been the preferred material for urethral reconstruction in patients with hypospadias. However, many studies, including those at our center, have found that there are still several differences in tissue composition (such as elastic fibers and smooth muscle components) between the urethral plate of hypospadias patients and the normal urethra [12, 13]. Nevertheless, it still has a more abundant blood supply and vigorous tissue vitality than other tissue because of its corpus cavernosum nature, so it’s relatively suitable to be used to reconstruct the urethra or serve as a part of the neourethra [14, 15]. For this reason, we preserved the distal and proximal urethral plates after transecting it at the point of the maximal curvature and then embedded the island flap into both ends of the urethral plates with a “V” shape, ensuring a spoon-shaped anastomosis at both ends. This maneuver not only makes the urethral plate part of the anastomosis but also increases the width of the tissue and, thus, the diameter of the neourethra, further reducing the possibility of postoperative anastomotic stricture. Of note, it can be observed that the postoperative stricture rate in the modified TPIF repair was significantly lower than that in the traditional TPIF procedure (4.65 vs. 10.81%) and even close to that in the two-stage procedure (3.23%). Thus, we conclude that this modification has a definite effect on reducing postoperative strictures. In addition, there were two cases of glanular urethral stricture with the modified TPIF repair. This may result from compression by external dense granular tissue, especially when the glanular diameter in the proximal hypospadias is generally small (both cases had narrow glans of < 8 mm width), with a more pronounced pressure. Therefore, it has also been suggested that for hypospadias with small glans, the neourethra can be temporarily made to the level of the coronal sulcus, and the distal urethra can be reconstructed after the glans grow.

A urethral diverticulum is another challenging complication of TPIF. The two most common causes are distal urethral strictures and urodynamic changes caused by tortuous or uneven neourethral diameter. In addition to meatus stenosis after the TPIF procedure, it should be noted that the neourethra distorts the lumen due to the lack of support in the corpus penis, resulting in a relatively high incidence of postoperative diverticulum. Encouragingly, our method also has certain advantages in preventing urethral diverticula. Specifically, the urethral plate is a relatively fixed tissue, with the remaining urethral plate proximally connected to the normal urethral tissue, distal to the tip of the glans. The neourethra were sutured on these two relatively fixed tissues, with both ends firmly anchored on the surface of the corpus cavernosum after penile straightening. In addition, unlike the traditional TPIF procedure, our maneuver involves tacking the flap on the proximal and distal urethral plate first, then trimming and tubularizing the flap longitudinally. This ensures that the length and width of the neourethra match the penis and guarantees that the neourethra will not be distorted and displaced, eventually reducing the possibility of diverticulum caused by poor urethral compliance after surgery. Although the incidence of diverticulum in the three groups was similar to that observed in our results, the probability of diverticulum in modified TPIF repair (2.33%) remained significantly lower than that (~ 12.8%) after traditional TPIF in the literature [11], which supports the validity of our method in improving urethral compliance.

No significant differences were found among the three groups regarding the incidence of postoperative urethrocutaneous fistulas. Technically, one difference between our method and the traditional TPIF procedure is that the suture line of the urethral tubularization is on the ventral side instead of the dorsal urethra clinging to the tunica albuginea, which is more similar to Byar’s procedure. From the actual results, however, no matter whether the suture line is on the dorsal or ventral side, it has little effect on the incidence of urethrocutaneous fistula. Therefore, the covering layers are more important to prevent the occurrence of urethrocutaneous fistulas.

Byar’s procedure, a classic two-stage method, pays more attention to the blood supply to the neo-urethra. Therefore, we chose Byar’s procedure as a control for this study. Our results demonstrated that the incidence of urethral stricture and urethrocutaneous fistula in Byar’s procedure is slightly lower than that in modified TPIF repair. Nevertheless, this difference cannot adequately indicate the superiority of the two-stage procedure compared to one-stage urethroplasty. Given several factors, such as treatment period, cost, and psychology, it is believed that many patients and surgeons will still choose a one-stage procedure. Therefore, one-stage repair of proximal hypospadias should still be a direction for clinicians.

Our study was limited by its retrospective nature, inevitable selection bias, and statistical error. Moreover, this was a single-center study with a limited number of patients. Some patients with short-term follow-up cannot temporarily manifest all complications. Moreover, many patients are too young to collect and compare urodynamic measures, such as uroflow rate, which will be completed by more cases and follow-up data in future prospective studies.

## Conclusion

Modified TPIF repair can ensure a wedge or spoon anastomosis between the proximal urethral meatus and the neourethra and provide support and blood supply for the neourethra. Furthermore, it extended the urethral plate width at the anastomosis and urethral meatus, effectively reducing the incidence of postoperative urethral strictures.

## Data Availability

The datasets generated or analyzed during this study are available from the corresponding author on reasonable request.

## Abbreviation

TPIF: Transverse preputial island flap
